# Drug quality analysis of isometamidium chloride hydrochloride and diminazene diaceturate used for the treatment of African animal trypanosomosis in West Africa

**DOI:** 10.1186/s12917-018-1633-7

**Published:** 2018-11-20

**Authors:** Zakaria Bengaly, Sèna Hervé Vitouley, Martin Bienvenu Somda, André Zongo, Assiongbon Têko-Agbo, Giuliano Cecchi, Yahaya Adam, Issa Sidibé, Balé Bayala, Adrien Marie Gaston Belem, Jan Van Den Abbeele, Vincent Delespaux

**Affiliations:** 1 Centre International de Recherche-Développement sur l’Elevage en zone Subhumide (CIRDES), Bobo-Dioulasso, 01 BP 454 Burkina Faso; 2 Université Nazi BONI (UNB), Bobo-Dioulasso, 01 BP 1091 Burkina Faso; 3 Département de Santé Publique-Environnement, Service de Pharmacie-Toxicologie, Ecole Inter-Etats des Sciences et Médecine Vétérinaires de Dakar (EISMV), Laboratoire de contrôle des médicaments vétérinaires (LACOMEV), BP 5077 Dakar-Fann, Sénégal; 4 Food and Agriculture Organization of the United Nations, Animal Production and Health Division, Rome, Italy; 5 Pong-Tamale Veterinary Station, Post Office Box 97, Tamale, Ghana; 6 Insectarium de Bobo-Dioulasso-Campagne d’Eradication de la mouche Tsé-tsé et des Trypanosomoses (IBD-CETT), Bobo-Dioulasso, 01 BP 1087 Burkina Faso; 7 Université de Ouaga I Pr Joseph KI-ZERBO, UFR/SVT, Ouagadougou, 03 BP 7021 Burkina Faso; 8 Biomedical Sciences Department, Institute of Tropical Medicine, Nationalestraat 155, B-2000 Antwerp, Belgium; 9 Bioengineering Department, Vrije Universiteit Brussel, Pleinlaan 2, B-1050 Brussels, Belgium

**Keywords:** African animal Trypanosomosis, Trypanocides, Drug quality, West Africa

## Abstract

**Background:**

Diminazene diaceturate (DA) and isometamidium chloride hydrochloride (ISM) are with homidium bromide, the main molecules used to treat African Animal Trypanosomosis (AAT). These drugs can be purchased from official suppliers but also from unofficial sources like local food markets or street vendors. The sub-standard quality of some of these trypanocides is jeopardizing the efficacy of treatment of sick livestock, leading thus to economic losses for the low-resource farmers and is contributing to the emergence and spread of drug resistance. The objective of this study was to assess the quality of trypanocidal drugs sold in French speaking countries of West Africa. In total, 308 drug samples including 282 of DA and 26 of ISM were purchased from official and unofficial sources in Benin, Burkina Faso, Côte d’Ivoire, Mali, Niger and Togo. All samples were analysed at LACOMEV (Dakar, Senegal), a reference laboratory of the World Organisation for Animal Health, by galenic inspection and high performance liquid chromatography.

**Results:**

The results showed that 51.90% of the samples were non-compliant compared to the standards and were containing lower quantity of the active ingredient compared to the indications on the packaging. The non-compliances ranged from 63.27% in Togo to 32.65% in Burkina Faso (61.82% in Benin, 53.84% in Mali, 50% in Côte d’Ivoire, 47.36% in Niger). The rates of non-compliance were not statistically different (*P* = 0.572) from official or unofficial suppliers and ranged from 30 to 75% and from 0 to 65% respectively. However, the non-compliance was significantly higher for ISM compared to DA (*P* = 0.028).

**Conclusions:**

The high non-compliance revealed in this study compromises the efficacy of therapeutic strategies against AAT, and is likely to exacerbate chemoresistance in West Africa. Corrective actions against sub-standard trypanocides urgently need to be taken by policy makers and control authorities.

## Background

In the 8 member countries of the “*Union Economique et Monétaire Ouest Africaine*” (UEMOA) (i.e. Benin, Burkina Faso, Côte d’Ivoire, Mali, Niger, Senegal, Togo, Guinea Bissau), livestock breeding and agriculture represent 30% of the Gross Domestic Product (GDP) and provide employment for 50% of the active population [1]. In particular, the livestock sector plays a key role in the economies of West African countries by providing 44% of the agricultural GDP, which rises to 50% if the work force and organic fertilizer are included [2]. In this context, reducing the constraints to agriculture that are posed by endemic animal diseases such as African trypanosomosis is of paramount importance for achieving food security and an alleviation of the poverty [3–5]. Strategies for limiting the impacts of trypanosomosis on animal health should target the vector, the parasite and the hosts. An ambitious continental programme aims at the creation and progressive expansion of tsetse and trypanosomosis-free areas i.e. the African Union - Pan African Tsetse and Trypanosomosis Eradication Campaign (AU-PATTEC), whose implementation started in Ethiopia, Kenya, Uganda in East Africa and Mali, Ghana, Burkina Faso in West Africa [6, 7]. However, tsetse flies are still widespread in West Africa, including in Burkina Faso [8, 9] and Mali [10], and unfortunately, the development of a vaccine against trypanosomosis remains elusive [11–13]. Thus, the control of the disease, currently, is heavily reliant on chemotherapy and prophylaxis. Three main molecules of trypanocides existing on the markets are: isometamidium chloride (ISM), diminazene aceturate (DA) and homidium salts, which represent respectively 40%, 30% and 26% of the total trypanocidal drug market by value [14]. However, homidium salts are no longer advisable since the discovery of their mutagenic effects [15].

Since the 90’s, the privatization and liberalization of the veterinary services led to a situation where veterinary drugs are more often supplied by untrained sellers and administered by the farmers or poorly experienced/trained extension workers [16, 17]. The sub-standard quality of drugs is frequently cited as one of the main factors contributing to the development and the spread of trypanocidal drug resistance [18]. A reinforcement and harmonisation of the veterinary legislation started in 2006 in the UEMOA zone and resulted in the creation of a unique marketing authorisation [19]. However, a recent study showed a high proportion of trypanocides of sub-standard quality purchased from both official and unofficial suppliers in Northern Togo [20].

The objective of this study was to assess the quality of trypanocidal drugs sold by official and unofficial suppliers in 6 French speaking countries (i.e. Benin, Burkina Faso, Côte d’Ivoire, Mali, Niger, Togo), except for Ghana and Nigeria that form the 8 member countries of RESCAO (*Réseau d’épidemiosurveillance de la chimiorésistance aux trypanocides et acaricides en Afrique de l’Ouest*) [21].

## Methods

### Study area

**Fig. 1:**
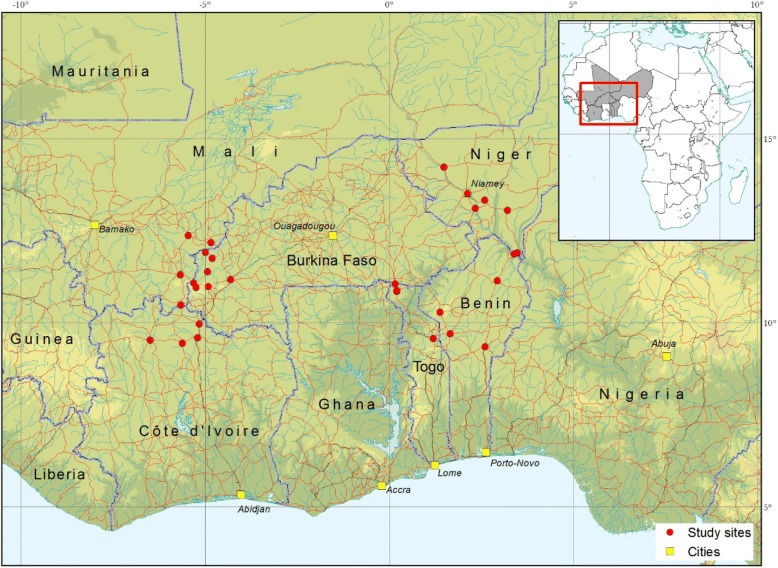
Sites of purchase of trypanocidal drugs in West Africa

The study was carried out in selected sites in Benin (5), Burkina Faso (7), Côte d’Ivoire (4), Mali (4), Niger (6) and Togo (4) (Fig. 1). In each country, samples were collected in the cotton-belt and pastoral areas characterized by an intense use of veterinary drugs [22, 23].

### Sample collection

Field protocol for sample collection was designed and provided by the *Laboratoire de contrôle des médicaments vétérinaires* (LACOMEV), a reference laboratory of the World Organisation for Animal Health (OIE), based in Dakar (Senegal). For each analysis, 10 sachets of 2.36 g or 5 sachets of 23.6 g DA and 10 sachets of 125 mg or 5 sachets of 1 g ISM were collected. Drugs were purchased in 2013 from official and unofficial sources, sealed in plastic bags and uniquely identified. Information available on the packaging (expiry date, manufacture date, etc.) was recorded in a standardized form. All the samples were purchased in dry galenic form except one liquid DA sample.

A total of 308 samples (282 of DA and 26 of ISM) were purchased out of which 98 and 210 from official and unofficial sources, respectively (Table 1). These samples of trypanocidal drugs included 62 trade names from 9 producing countries (Belgium, China, England, France, Germany, Netherlands, India, Nigeria and Spain) for DA and 6 trade names from 2 countries (France and Netherlands) for ISM.

**Table 1:** Distribution of samples per country

Country	Sample’s names	Sources		Total
		Unofficial circuit	Official circuit	
Benin	DA	44	8	52
	ISM	3	0	3
Burkina Faso	DA	49	1	50
	ISM	4	0	4
Côte d’Ivoire	DA	35	11	46
	ISM	3	3	6
Mali	DA	14	35	49
	ISM	3	2	5
Niger	DA	22	16	38
	ISM	1	4	5
Niger	DA	32	15	47
	ISM	0	3	3

Legend: *DA* diminazene diaceturate, *ISM* isometamidium chloride hydrochloride

All samples collected from the field were stored away from heat and moisture at CIRDES (*Centre International de Recherche-Développement sur l’Elevage en zone Subhumide*) in Bobo-Dioulasso (Burkina Faso), then labelled and packaged before shipment to LACOMEV for subsequent analysis.

### Trypanocide quality analysis

All the drugs collected were analysed at LACOMEV prior to the expiry date indicated on the packaging, except for 19 samples (16 DA and 3 ISM) which were expired. The quality was determined firstly by galenic test including pH measurement, the solubility of the DA liquid as well as solutions reconstituted from DA or ISM granules according to the manufacturer’s recommendations. Secondly, the active ingredient was identified and its concentration measured using high performance liquid chromatography (HPLC). The galenic inspection and HPLC were carried out according to the OIE’s monograph prepared by the consortium GALVmed/FAO/IAEA/IFAH [15] using reference samples. A pH between 4 and 7 was considered as compliant. The solubility of solutions (liquid or reconstituted) was visually assessed with the naked eye for the absence of solid particles.

**Fig. 2:**
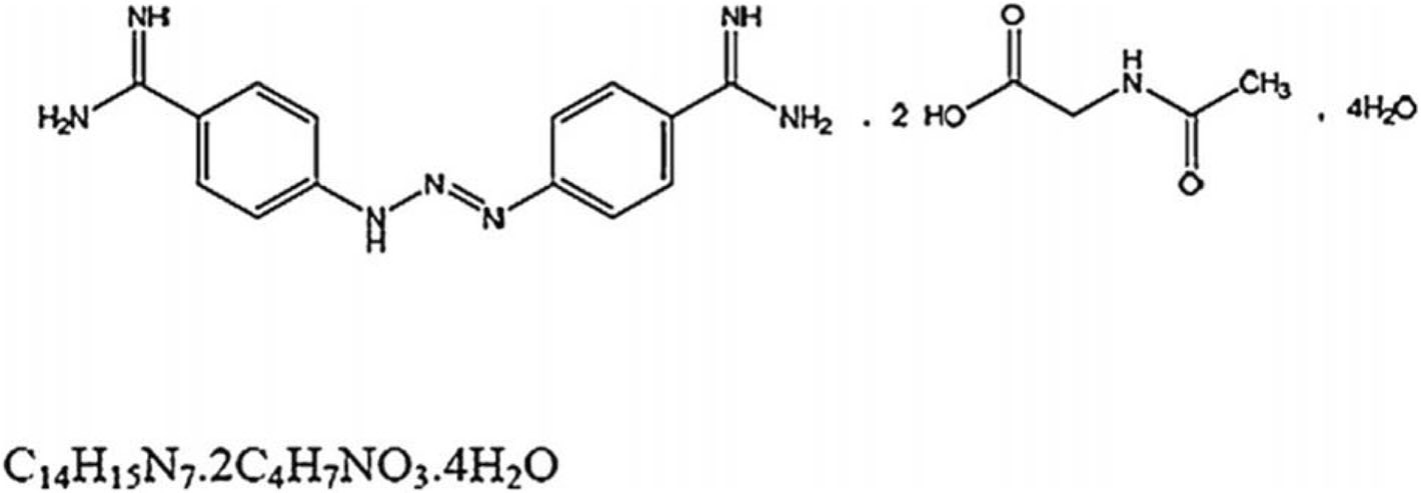
Molecular structure of diminazene diaceturate [13]

**Fig. 3:**
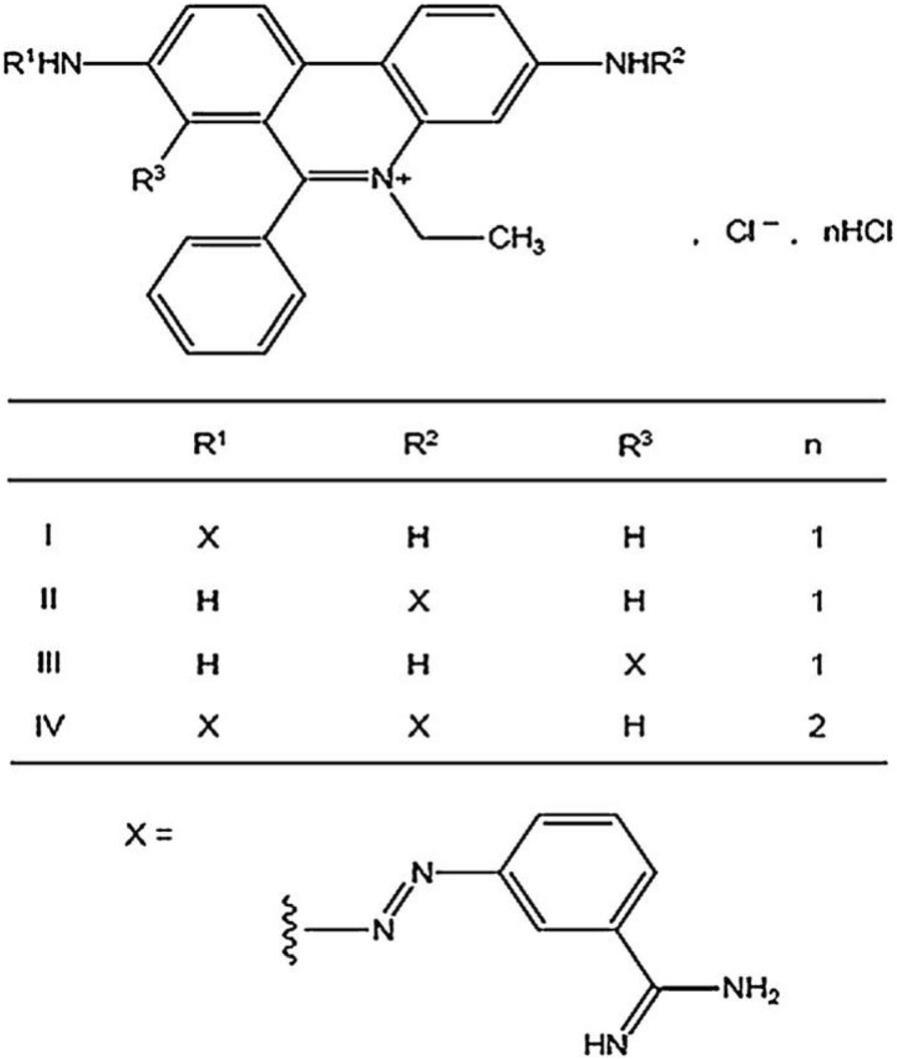
Molecular structure of isometamidium chloride hydrochloride [13]

For DA, a measured concentration within ±10% of the manufacturer’s label claim was considered as compliant according to the threshold applied by LACOMEV [24]. For ISM, the following criteria according OIE monograph were used: (i) presence of the four isomers (I, II, III, IV), (ii) a proportion of isomer I (principal component) equal to or greater than 55%, (iii) a proportion of isomers II, III and IV was equal to or less than 40% and (iv) a proportion of the four isomers between 95 and 102% (Figs. 2 and 3).

### Data analysis

All statistical analyses were performed with the JMP 11 software (SAS Institute Inc.). The chi^2^-test was used to compare the different proportions of non-compliance. A logistic regression model was built using country, source (official or not) and compound (DA or ISM) as explanatory variables to determine the factors involved in the non-compliance of the trypanocidal drugs. *P-values* (*P*) < 0.05 were considered significant.

## Results

### Proportion of non-compliant samples by country

**Fig. 4:**
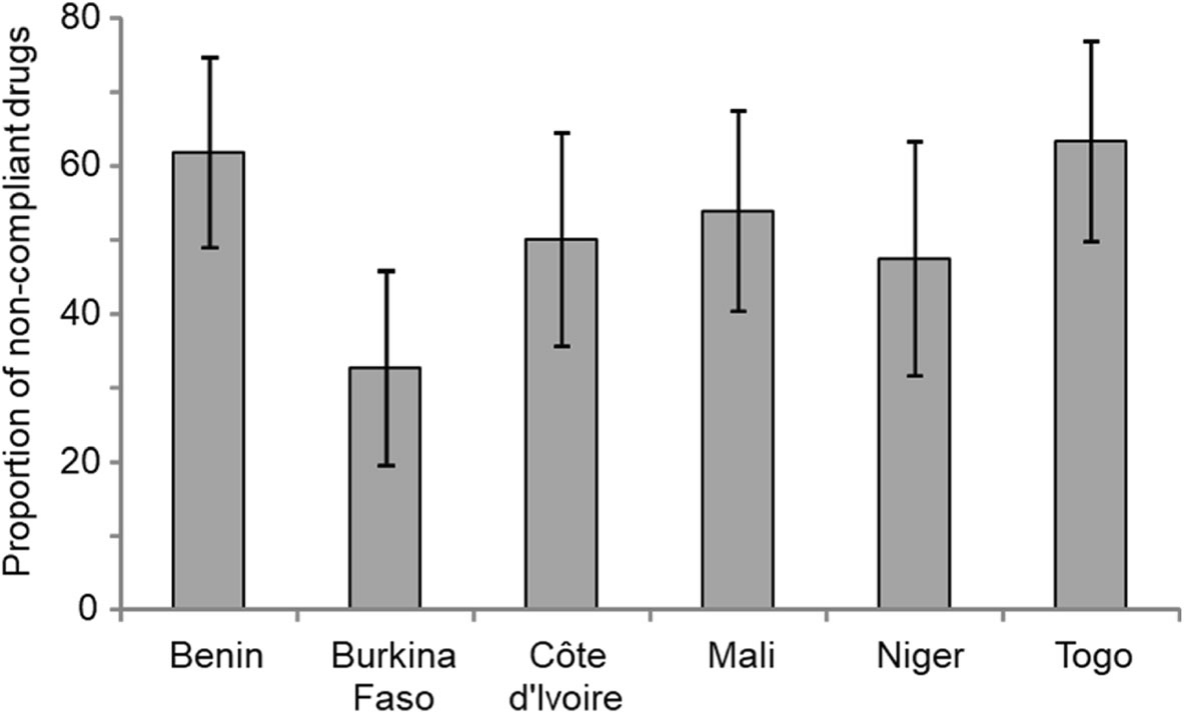
Proportion of non-compliant trypanocides by country. All proportions were different between countries (chi^2^-test, *P* = 0.025). On histograms, vertical bars (above and below) represent 95% confidence intervals of the proportion

The 19 samples (16 DA and 3 ISM) which were expired, were not included in analysis. The galenic and the HPLC tests allowed defining the compliance or non-compliance status of the other samples. The overall proportion of non-compliance of these was 51.90%. The proportions differed between countries (*P* = 0.025), with the lowest in Burkina Faso (32.65%) and the highest in Togo (63.27%). The proportions of non-compliance by country are illustrated in Fig. 4.

### Proportion of non-compliance by source

**Fig. 5:**
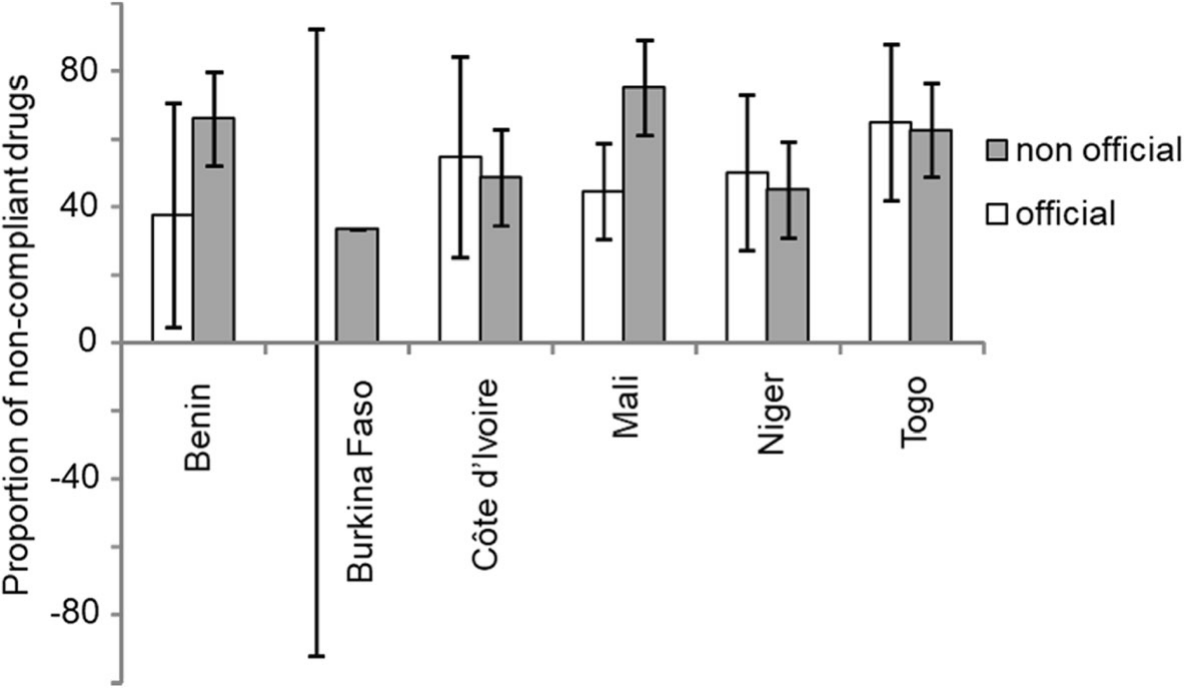
Proportion of non-compliant trypanocides by source. No difference was highlighted between unofficial and official sources for purchasing of trypanocidal drugs (chi^2^-test, *P* = 0.572). Vertical bars (above and below) on histograms represent 95% confidence intervals of the proportion

No significant difference in non-compliance between the two supply sources was identified (Fig. 5). In the unofficial market 53.03% of the samples were non-compliant, against the 49.45% in the legal market (*P* = 0.572). However, in Mali, the rate of non-compliance was significantly higher in samples from the unofficial market compared to the official ones (75% vs 44.44%, *P* = 0.0414). These rates were not significantly different in Benin (37.5% vs 65.96%, *P* = 0.1256), in Côte d’Ivoire (54.55% vs 48.57%, *P* = 0.7296), in Niger (50% vs 45%, *P* = 0.7579) and in Togo (64.71% vs 62.5%, *P* = 0.8788). In Burkina Faso, only 1 DA sample was collected from the official market and found to be compliant by LACOMEV, whereas 33.33% of the samples from the unofficial market were found to be non-compliant.

### Proportion of non-compliance by active ingredient (Fig. 6)

The results showed a significantly higher non-compliance (*P* = 0.028) with ISM (73.91%) compared to DA (50%). However, no significant difference was observed between the two sources of supply (*P* = 0.057 and *P* = 0.178 for the official and unofficial sources, respectively). Logistic regression using the country of purchase, the source (official or not) and active ingredient (ISM or DA) as explanatory variables revealed statistically significant effects of the active ingredient (*P* = 0.014) and country (*P* = 0.013) (Table 2).

**Fig. 6:**
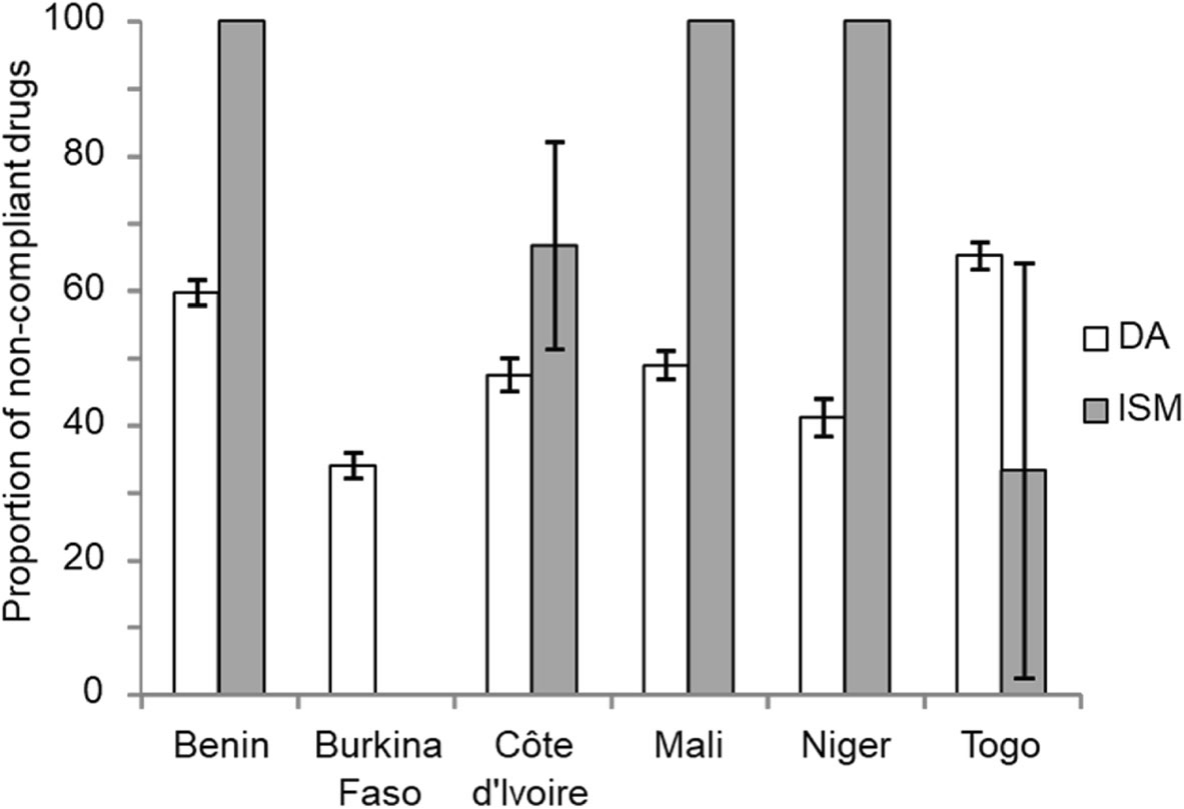
Proportion of non-compliant trypanocides. The non-compliance between diminazene diaceturate and isometamidium chloride hydrochloride was significantly different (chi^2^-test, *P* = 0.028). On histograms, vertical bars (above and below) represent 95% confidence intervals of the proportion

**Table 2:** Sampling country, source and active ingredient effects on non-compliance of trypanocides in West Africa

Covariates		chi2 ± Standard Deviation	*p* ^a^
Country of purchase	Benin	1.99 ± 0.26	**0.013**
	Burkina Faso	9.41 ± 0.29	
	Côte d’Ivoire	0.34 ± 0.28	
	Mali	0.86 ± 0.29	
	Niger	0.19 ± 0.30	
	Togo	4.10 ± 0.27	
Source		2.63 ± 0.15	0.111
Active ingredient		5.32 ± 0.25	**0.014**

Logistic regression model of non-compliance built with the country of sampling, source (official or not) and active ingredient (DA or ISM) as explanatory variables. ^a^*P-values* < 0.05 are bolded

## Discussion

The use of good quality trypanocidal drugs is required for an efficient treatment of AAT and for preventing the emergence of chemoresistance [25]. It is also considered as a key component for sustainable, community-based AAT reduction in the framework of the Progressive Control Pathway for AAT [3]. This study provides data on the quality of trypanocides in 6 French speaking, which are members of the RESCAO network in West Africa.

The high proportion of non-compliance obtained in the sub-region (51.90%) shows that trypanocidal drugs of sub-standard quality remain a major reason of concern. It should also be considered here that the current threshold of deviance for DA used by LACOMEV and for this study was ±10% [24], while the threshold recommended by the OIE is more stringent being only ±5% in the routine analyses of drug control laboratories [15]. This means that the non-compliance would be higher than 51.90%, reaching approximately 76% with the threshold of ±5%.

Non-compliances were significantly different between countries and when compared to previous studies using the same ±10% threshold, the non-compliances were of the same order of magnitude: 32.63% vs 42.3% in Burkina Faso [26]; 50% vs 71.4% in Côte d’Ivoire [27] and 63.27% vs 40% in Togo [20]. The same proportions of high non-compliances were observed in Senegal [28] and Cameroon [29].

Our study also showed that non-compliances were similar from the official and the unofficial sources of supply (49.45% vs 53.03%). The high proportion of non-compliance might not be explained only by poor conditions of storage, transport and/or handling, but probably also by a poor quality control during manufacturing [20]. The lower price of the trypanocides sold in the unofficial market is particularly obvious by the weekly markets in villages, which are more visited by livestock keepers. According to the International Federation for Animal Health, the trade in unregistered and substandard veterinary drugs in Africa is worth $400 million annually - a size similar to the official market [30].

In the RESCAO network area, a significantly higher non-compliance was observed for ISM (73.91%) compared to DA (50%), but no difference was detected between the supply sources (official or unofficial). The non-compliance rates were heterogeneous between countries and observed for both for DA and ISM. In Togo, non-compliances were in agreement with the results of a recent study: 33.33% vs 19% for ISM and 65.22% vs 50% for DA [20]. The regression model confirms that this non-compliance is linked both to the active ingredient and to the country. Despite the mandatory marketing authorisation delivered by the UEMOA for any veterinary drugs, more actions are needed to reinforce the control and the sensitization of the sellers and actors of the livestock breeding in each country. These actions will help to reduce illegal trade of poor quality trypanocides, and thus to prevent or reduce drug resistance in the RESCAO network area.

## Conclusion

The results of this study on the quality of trypanocides showed a high proportion of non-compliant drugs used for the treatment of AAT in West Africa. This study did not take into account the presence of contaminants or impurities and the authenticity of trypanocidal drugs. This non-compliance compromises the efficacy of the treatment as well as boosts the development of drug resistance, which is already observed in several African countries. To limit the deleterious effects of the circulating sub-standard quality drugs, the following actions would be, through UEMOA: (i) to enforce the national and sub-regional regulations and laws on the quality control, importation, supply and use of veterinary medicines and (ii) to organise capacity building of veterinary personal and livestock farmers on a rational use of the available drugs.

## Abbreviations

AAT: African animal trypanosomosis
CIRDES: Centre International de Recherche-Développement sur l’Elevage en zone Subhumide
DA: Diminazene aceturate
FAO: Food and Agriculture Organization
GALVmed: Global Alliance for Livestock Veterinary Medicine
GDP: Gross domestic product
HPLC: High performance liquid chromatography
IAEA: International Atomic Energy Agency
IFAH: International Federation for Animal Health
ISM: Isometamidium
LACOMEV: Laboratoire de Contrôle des Médicaments Vétérinaires
OIE: World Organisation for Animal Health
RESCAO: Réseau d’épidemiosurveillance de la chimiorésistance aux trypanocides et acaricides en Afrique de l’Ouest
UEMOA: Union Economique et Monétaire Ouest Africaine
